# Directional Resonant MEMS Acoustic Sensor and Associated Acoustic Vector Sensor

**DOI:** 10.3390/s23198217

**Published:** 2023-10-01

**Authors:** Justin Ivancic, Gamani Karunasiri, Fabio Alves

**Affiliations:** Department of Physics, Naval Postgraduate School, Monterey, CA 93943, USA; justin.ivancic@nps.edu (J.I.); gkarunas@nps.edu (G.K.)

**Keywords:** MEMS acoustic sensor, MEMS acoustic vector sensor, resonant sensor

## Abstract

This paper reports on the design, modeling, analysis, and evaluation of a micro-electromechanical systems acoustic sensor and the novel design of an acoustic vector sensor array (AVS) which utilized this acoustic sensor. This research builds upon previous work conducted to develop a small, lightweight, portable system for the detection and location of quiet or distant acoustic sources of interest. This study also reports on the underwater operation of this sensor and AVS. Studies were conducted in the lab and in the field utilizing multiple acoustic sources (e.g., generated tones, gun shots, drones). The sensor operates at resonance, providing for high acoustic sensitivity and a high signal-to-noise ratio (SNR). The sensor demonstrated a maximum SNR of 88 dB with an associated sensitivity of −84.6 dB re 1 V/μPa (59 V/Pa). The sensor design can be adjusted to set a specified resonant frequency to align with a known acoustic signature of interest. The AVS demonstrated an unambiguous, 360-degree, in-plane, azimuthal coverage and was able to provide an acoustic direction of arrival to an average error of within 3.5° during field experiments. The results of this research demonstrate the potential usefulness of this sensor and AVS design for specific applications.

## 1. Introduction

The design, modeling, analysis, and evaluation of a micro-electromechanical systems (MEMS) directional acoustic sensor operating at resonance and an associated acoustic vector sensor (AVS) is presented. Determining the direction of arrival (DOA) of sound has long been an active field of study in acoustics. The DOA can be determined via many different devices and techniques.

### 1.1. Biologically Inspired Sensors

In the field of MEMS vector sensors, significant inspiration has been drawn from biology. Many MEMS acoustic sensors have been designed based on the hearing organs of humans as well as certain lizards, mosquitos, locusts, and flies [1]. The acoustic device presented here draws inspiration from the hearing organ of a fly.

In 1995 Miles et al. [2] described the hearing organ of the fly, *Ormia ochracea*, and how the fly is able to determine the DOA of sound. This hearing organ consists of two mechanically coupled tympana (eardrums). In 2006 Arthur and Hoy [3] demonstrated in lab conditions that the fly could reliably navigate towards sounds of interest. In 2008 Akcakaya and Nehorai [4] analyzed the physical performance of the ear with respect to DOA estimation and since then, various MEMS acoustic sensors, inspired by *Ormia ochracea*, have been investigated [5,6,7,8,9,10,11,12,13,14,15,16,17,18,19,20,21,22,23,24,25,26,27,28]. In general, these designs consisted of mechanically coupled vibrating membranes and a method to sense the vibration. The membrane vibration was typically sensed with capacitive circuits, piezoelectric arms, or optical sensors.

### 1.2. Directional Sensors

The most important factor to *Ormia ochracea* inspired sensors is that these sensors typically exhibited a cosine-like directionality, where a maximum response is detected with the DOA normal to the sensor surface. This response drops sinusoidally to zero as the DOA is rotated by ninety degrees [29].

Ishfaque et al. [12], in 2019, described a circular membrane MEMS sensor with a piezoelectric sensing system. This research investigated methods to minimize noise levels and to maximize the signal-to-noise ratio (SNR). They reported a sensitivity of −167 dB re 1 V/μPa (4.36 mV/Pa) at 1 kHz and an SNR of 66.77 dB. In 2019 Rahaman and Kim [13] demonstrated an AVS which consisted of two MEMS sensors aligned orthogonally. The DOA of incoming sound (limited to a single quadrant) was calculated using an arctangent algorithm. While a graph comparing measured and actual DOA was presented, no explicit DOA accuracy was discussed. Rahaman and Kim [15] presented a different AVS design in 2020 consisting of two coupled wing-like diaphragms. They employed an arccosine function to determine DOA with 180-degree azimuthal coverage and average error of 2.6 degrees. The SNR of the sensors was reported to be about 68.5 dB. In 2021, Rahaman et al. [16] demonstrated a double-wing-designed sensor utilizing a piezoelectric readout with a sensitivity of −139 dB re 1 V/μPa (110.5 mV/Pa) measured at 1 kHz. This sensitivity measurement was taken at a frequency significantly below the sensor’s primary eigenmodes. Ren and Qi [17], in 2021, reported on a double-winged sensor that utilized a laser to measure the wing deflection, and showed a low noise floor and highly repeatable sound pressure measurements. Also in 2021, Shen et al. [18] described an *Ormia ochracea*-inspired sensor utilizing an intermembrane bridge. The sensor consisted of two separate circular membranes mechanically coupled by a structure that pivoted between the two membranes. They reported a theoretical acoustic DOA resolution of two degrees with an optimal frequency range of 300 Hz to 1500 Hz. In 2022 Rahaman and Kim [19] described an array of three double-wing sensors collocated and arranged at 120-degree angles to each other. They reported the angular resolution of the sound source localization to be ±2 degrees in the horizontal azimuth and elevation; however, localization required a priori knowledge of one or the other. The reported SNR of the sensor was 66.77 dB with a sensitivity of −167 dB re 1 V/μPa (4.36 mV/Pa).

### 1.3. Resonant Sensors

Another key aspect of the sensor presented in this paper is that it is designed to operate at, or near, resonance. Typically, microphones are designed to have a relatively constant sensitivity over a large frequency range. Sensors that are designed to operate near resonance trade increased performance at the cost of a reduced frequency range. In applications where tonal detection or signature-based harmonic detection is desired, resonant acoustic sensors can be designed with the advantage of mechanically filtering noise outside of the frequency range of interest [30,31,32,33,34,35,36].

In 1998 Schoess et al. [31] demonstrated a simple, resonant, integrated microbeam sensor for detecting pending mechanical failure in aircraft components. The reported SNR of the sensor was 6:1 (15.6 dB) and operated at a resonant frequency of 312 kHz.

In 2017, Kusano et al. [37] demonstrated a small-sized yet low-frequency (430 Hz), 3D-printed, spiral-shaped resonator based on the human cochlea. This resonator was coupled with a commercial MEMS microphone to create a low-power system tuned to detect acoustic frequencies of interest. They demonstrated a 9 dB amplification in sound pressure at the fundamental mode of the resonator with an acoustic sensitivity of up to −154.4 dB re 1 V/μPa (19 mV/Pa).

Although not a MEMS sensor, in 2020 Lee et al. [35] presented a resonant acoustic sensor for DOA determination using acoustically coupled Helmholtz resonators. In 2020 Li et al. [36] presented a 210-beam sensor. The research explored the optimization of a silicone-layer thickness for acoustic sensitivity enhancement, reporting multi-cantilever sensitivity of 72 V/m/s (sensitivity measured in terms of output voltage to unit velocity).

### 1.4. Combined Resonant and Directional Sensors

The sensor presented in this paper follows years of research at the Naval Postgraduate School. Touse et al. [20,21], in 2010, demonstrated a double-wing sensor design. This design featured interdigitated comb fingers between the ends of the wings and a substrate enabling a capacitive readout. The research investigated the cosine-like directionality of the sensor as well as geometric design options to emphasize particular vibration modes. In 2014, Downey and Karunasiri [22] investigated device-layer thickness and comb finger design effects on the sensors’ acoustic sensitivity and overall wing displacement. In 2016, Wilmott et al. [23] developed an AVS using two double-wing sensors canted with a 30-degree offset. This design moved towards reducing the azimuthal ambiguity of an AVS. At a resonance frequency of 1.69 kHz, a sensitivity of −92.0 dB re 1 V/μPa (25 V/Pa) was measured. The AVS was able to determine the DOA to within a 3.4-degree accuracy over a ±60-degree arc. In 2020, Espinoza et al. [24] presented an investigation of a similar sensor in an underwater environment. Two sensors were presented, a double-wing and single-wing design. The sensors were placed in a housing filled with silicone oil. Underwater sensitivity for both sensors was approximately −165 dB re 1 V/μPa (6 mV/Pa) measured at resonance (125 Hz single wing, 242 Hz double-wing). Both sensors demonstrated a cosine-like directivity pattern. 

Rabelo et al. [25], in 2020, demonstrated how separating a sensor’s output signal into a superposition of rocking and bending modes, and determining the phase shift between them, could be used to calculate the DOA over a 180-degree arc. In 2022, Alves et al. [26] presented another underwater version of the sensor. The sensor was enclosed in a near neutrally buoyant, air-filled housing. At resonance (1.6 kHz), the sensitivity was −149 dB re 1 V/μPa (37 mV/Pa) with an SNR of about 38 dB. The sensor displayed a dipole directionality pattern that was more cosine-like than previous underwater designs. Again in 2022, Alves et al. [27] demonstrated double-wing sensors where each wing had a different resonant frequency (e.g., left wing: 718 Hz, right wing: 658 Hz). Combining the outputs of each wing was shown to broaden the frequency peak of the sensor. A sensitivity of −97.7 dB re 1 V/μPa (13 V/Pa) was measured with an average SNR of 91 dB for a band pass of 120 Hz. The sensor displayed a cosine-like directionality. Crooker et al. [28], in 2023, presented algorithms for calculating unambiguous 360-degree DOA. These algorithms account for minor differences in the individual acoustic sensors, used by the AVS, which would otherwise introduce DOA errors.

A common problem with the AVSs discussed above is the limitation in azimuthal range. Single sensors or a combination of them were not able to resolve 360 degrees of acoustic DOA without ambiguity or a priori knowledge of some parameters. The AVS design presented in this paper addresses these shortfalls. In this paper, we describe the design and characterization of a double-wing MEMS sensor, similar to the ones demonstrated in [23,27]. Using these MEMS sensors, we demonstrate a novel AVS design capable of non-ambiguous DOA determination over 360 degrees with a significantly higher SNR than similar sensors.

## 2. Design and Modeling

### 2.1. Design Requirements

Distant and quiet acoustic sources can be difficult to detect and track with conventional acoustic detectors. High SNR, directionality, small size, light weight, and low power consumption are sensor characteristics often required by many applications, particularly those associated with national defense. AVSs meeting these requirements can be made using two MEMS directional acoustic sensors operating at resonance [20,21,22,23,24,25,26,27,28] coupled with one omnidirectional microphone.

While the sensor described in this paper is inspired by the *Ormia ochracea*, there are significant differences between the fly and this sensor. The fly utilizes multiple vibration modes of its hearing organ to determine acoustic DOA with a single pair of mechanically coupled membranes. The sensor presented here utilizes only one vibration mode and calculates acoustic DOA using two MEMS sensors and an omnidirectional microphone.

### 2.2. Sensor Construction

**Figure 1:**
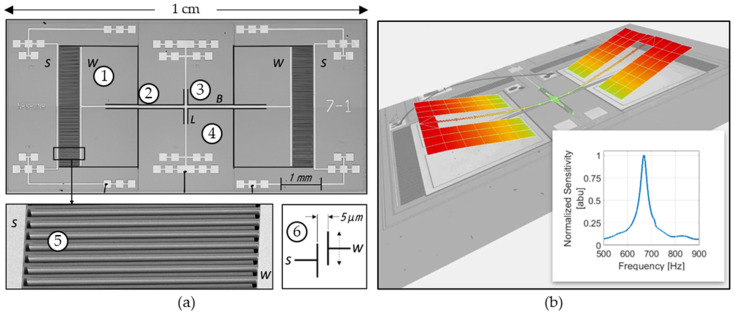
Image of MEMS sensor: (**a**) microscope image of MEMS sensor: (1) wing, (2) bridge, (3) torsional legs, (4) substrate, (5) SEM image of comb fingers (for capacitive sensing), (6) diagram showing the 5 μm gap between comb fingers; and (**b**) laser vibrometry image of MEMS sensor vibrating in bending mode. Inset: normalized sensitivity response of sensor showing a resonance at about 680 Hz.

The directional sensor presented in this paper is a MEMS device consisting of a pair of identical wings connected by a bridge, as shown in Figure 1. The wings vibrate out of the plane of the sensor substrate when exposed to acoustic waves. The bridge is anchored to the substrate by torsional legs that are aligned perpendicular to the bridge. In this sensor design, the torsional legs are located along the center of the bridge, making the sensor symmetrical. Interdigitated comb fingers are located between the ends of the wings and the substrate, allowing the displacement of the wings to be measured via a capacitance-sensing circuit.

When exposed to acoustic waves, the sensor is subject to a rocking mode (the wings move opposite to each other) and a bending mode (wings move with each other), as shown in Figure 1b. In this design, the back of the sensor is open to the environment, enhancing the bending mode and diminishing the rocking mode. The bending mode exhibits a cosine-like response to acoustic DOA. The sensor is designed to detect sounds with component frequencies near the resonance (~700 Hz), allowing for increased SNR in a narrow frequency band (FWHM ~25 Hz). This also reduces the effects of noise outside of the frequency band. The resonance and FWHM can be tailored by design by changing the size and shape of the sensor components. The impact of most of these design parameters translates into the stiffness of the beam, effective mass, characteristic length, and fluid density. Detailed parametric simulations are beyond the scope of this paper. Minor physical differences (due to microfabrication tolerances) in each sensor lead to observable differences in sensor responses. A detailed analysis of the fabrication imperfections is beyond the scope of this paper; however, differences in the mass of the wings due to under-etch or over-etch can cause changes in the resonant peak. Thinner or thicker comb fingers due to under- or over-etch can cause differences in the mass of the wings (changes in the resonant peak) and damping (changes in the quality factor). Microscope inspection reveals that minor differences exist from sensor to sensor (e.g., missing comb fingers, inconsistent gap etching between wing and substrate comb fingers, device-layer thickness). Laser vibrometry measurements of multiple sensors show an average resonant frequency of 671 Hz and average quality factor of 27. 

**Figure 2:**
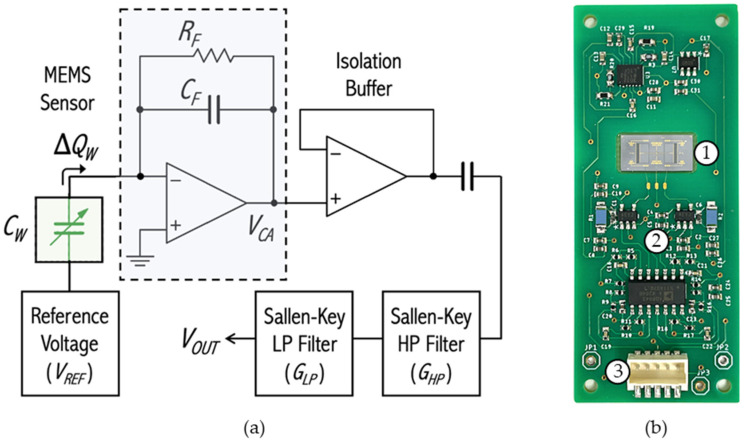
(**a**) MEMS sensor capacitive readout circuit diagram; and (**b**) MEMS sensor mounted in PCB: (1) MEMS sensor, (2) capacitive readout circuitry, (3) wired connection to supply power and to read sensor output.

The sensor is microfabricated by a commercial foundry (MEMSCAP) [38] on a 400 μm thick silicon-on-insulator (SOI) wafer with a 25 μm silicon device layer deposited on top of the wafer. The device layer includes gold contact pads which allow for the sensor to be wire-bonded to a printed circuit board (PCB) which contains a capacitance readout circuit, as shown in Figure 2.

Figure 2a shows a block diagram of the electronic readout. The charge amplifier circuit (dashed rectangle) includes an operational amplifier, which connects sensor and feedback capacitors to a high-impedance virtual ground at its inverting input. The variable capacitance *C_W_* represents the interdigitated comb finger capacitors of the MEMS directional sound sensor. Assuming that *C_W_* is biased under a constant voltage *V_REF_*, when the wings are at rest, an equilibrium position of the comb fingers’ overlap is achieved and the capacitance between them is constant. Consequently, the accumulated charge on its plates, *Q_W_*, is constant as well. Under these conditions, no electric current (ideally) flows to, or from, the op amp nor does it flow through the feedback network; the output voltage *V_CA_* follows the inverting input voltage, which in this case is virtual ground. When an acoustic pressure wave impinges on the sensor’s wings, their equilibrium position at rest is changed along with the overlap of comb fingers, resulting in a change in the wing capacitance to a new value, *C’_W_*. If the biasing voltage is kept constant, the accumulated charge in the comb finger capacitors must readjust to a new value, *Q’_W_*. The charge difference, ∆*Q_W_*, is pushed to, or pulled from, the remainder of the circuit. Since the op amp input impedance is much larger than that of the feedback capacitor, *C_F_*, all the charge variations from the sense capacitor, *C_W_*, move through *C_F_*, developing an output voltage, *V_CA_*, given by

(1) $$V_{CA}=-\frac{{∆Q}_{W}}{C_{F}}=-\frac{{∆C}_{W}}{C_{F}}V_{REF}.\;$$

Two Sallen–Key filter stages are added to limit the passband between 100 and 3500 Hz.

**Figure 3:**
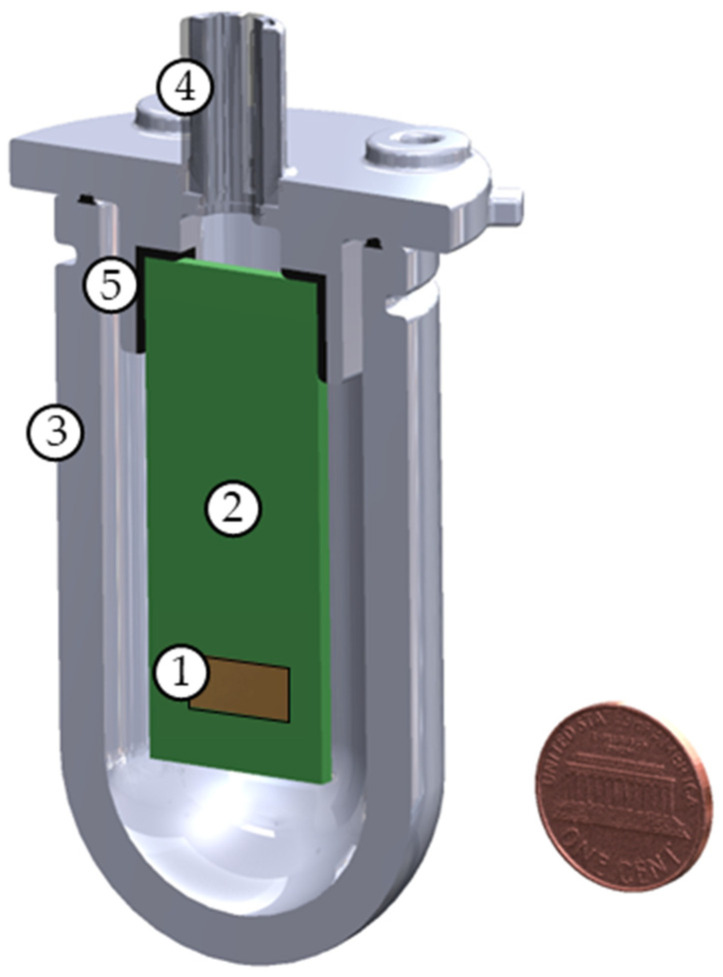
Planar sliced diagram of 3-D printed underwater housing with mounted PCB (penny included for sense of scale): (1) MEMS sensor, (2) PCB, (3) watertight sensor housing, (4) watertight wire penetration, and (5) bracket to mount PCB to housing.

The sensor is intended to be used both in air and underwater. For underwater operation, the sensor is installed in a smaller PCB with a similar readout circuit. The sensor and PCB are enclosed in an air-filled, watertight housing, shown in Figure 3. The housing is a 3-D printed cylinder constructed of plastic (Rigur, VeroWhitePlus) and rubber (AGILUS30 FLX935) [39]. This material was found to be acoustically opaque (~3.5% transmissivity) for the frequency range of the sensor. For underwater usage, the sensor is in a near neutrally buoyant housing. In this configuration, the housing vibrates with the same velocity as the particle velocity of the fluid that would result if the housing was removed [40]. When the sensor housing is exposed to an acoustic wave, the MEMS sensor behaves like an inertial sensor. The wings act as a proof mass while the substrate vibrates with the rest of the sensor housing.

### 2.3. Analytical Modeling

The sensor operation can be approximated as a driven, damped harmonic oscillator. The complexities in the shape of the sensor and various sources of damping make this design appropriate for finite element modeling (FEM). However, some simple assumptions and approximations allow for analytical methods to be used for the sensor design.

In the bending mode, the wings bend up and down together, causing no torque to be applied to the torsional arms. Analytical models of the sensor can be simplified to a single wing in a clamped-free configuration. The stiffness of the micro-scale sensor bridge matches that of larger-scale beams [41].

(2) $$k_{bridge}=\frac{Ewt^{3}}{4L^{3}}\;,$$

where E is Young’s modulus, *w* is the width of the beam, *t* is the thickness of the beam, and *L* is the length of the beam. The stiffness of the wing is much greater than the stiffness of the bridge, therefore the wing does not bend significantly compared to the bridge. Therefore, the sensor can be further simplified to a simple mass-loaded beam. In this model, the wing is treated as a point mass (*m_eff_*) located at the end of the bridge with an equivalent moment of inertia in the wing. The natural resonant frequency can then be modeled by

(3) $$\omega_{0}=\sqrt{\frac{k_{bridge}}{m_{eff}}}\;\;.$$

Resonating MEMS devices are subject to multiple damping effects. The significance of these damping sources depends strongly on the design of the device [42]. The damping caused by the fluid surrounding our sensor is modeled by accounting for two primary effects: the damping that exists due to a beam vibrating alone in a fluid and damping caused by the fluid flow between the sensor and the substrate. Sader [43] describes a method for modeling a cantilever beam of an arbitrary but uniform cross-section that vibrates while immersed in a viscous fluid. Our sensor does not have a uniform cross-section; however, Sader’s method was adapted to model it. Sader’s method calculates a Reynolds number using a characteristic length based on the width of the beam. Our model uses the width of the wing when calculating this Reynold’s number, given by

(4) $$Re=\frac{\rho \omega b^{2}}{4\mu }\;,$$

where *ω* is the vibrational frequency of the beam, *ρ* is the fluid density, *μ* is the fluid dynamic viscosity, and *b* is the characteristic length (width of the wing for our purposes). The Reynold’s number is used to calculate a hydrodynamic function (Γ), which is used to determine the resonant frequency and quality factor. Multiple equations are needed to calculate Γ, and are beyond the scope of this paper. Suffice to say, we used the hydrodynamic function to calculate the resonant frequency and quality factor using

(5) ω1ω0=1+πρb24μΓr−12,

and

(6) $$Q_{1}=\frac{\frac{4\mu }{\pi \rho b^{2}}+\Gamma_{r}}{\Gamma_{i}}\;,$$

where *ω*_1_ and *Q*_1_ are the resonant frequency and quality factor modeled by Sader’s method. Γ*_r_* and Γ*_i_* are the real and imaginary parts of the hydrodynamic function detailed in [43]. When the sensor operates in air, Sader’s method produces estimated resonant frequencies within 6% of the average measured values. However, when the sensor is placed in a more dense, less viscous fluid, Sader’s model by itself is insufficient.

Sader’s model assumes that the beam vibrates alone in the fluid. Our sensor is surrounded by a substrate. There is a narrow gap between the edges of the wing and substrate, and an even more narrow gap between the interdigitated comb fingers of the wing and substrate. Couette flow in these gaps was considered. The drag force from the Couette flow can be related to the mechanical resistance of a simple harmonic oscillator system [44,45] as

(7) $$R_{m}=\mu \left(\frac{A_{w}}{g_{w}}+\frac{A_{c}N}{g_{c}}\right),$$

where *R_m_* is the mechanical resistance. *A_w_*, *A_c_*, *g_w_*, *g_c_*, and *N* are the surface area on the sides of the wing, surface area on the side of a single comb finger, the gap distance between the wing and substrate, gap distance between comb fingers, and number of comb fingers on the wing, respectively. The Couette flow contribution to the quality factor of the sensor is then

(8) $$Q_{2}=\frac{m_{eff}\omega_{0}}{R_{m}}.$$

These two separate damping sources are accounted for in our analytical model by calculating a total quality factor, *Q_t_*, via

(9) $$Q_{t}={\left(\frac{1}{Q_{1}}+\frac{1}{Q_{2}}\right)}^{-1}.$$

A best fit curve for *Q* as a function of frequency is calculated based on *Q* → 0 as *ω* → 0, *Q* → ∞ as *ω* → *ω*_0_, and *Q*(*ω*_1_) = *Q*_1_. *Q_t_* is evaluated against this best fit curve to determine the resonant frequency, *ω_t_*.

To date, the sensor described in this paper (model 7-1) has been operated only in air (the sensor has been used in underwater applications while encased in an air-filled housing). However, a similar sensor (model 7-3) has been operated in air and in a low-viscosity silicone oil. Table 1 shows a comparison of the analytically modeled resonance frequency and quality factor against average values measured in laboratory conditions.

### 2.4. Finite Element Modeling

FEM analysis of the sensor designs was conducted using COMSOL Multiphysics software version 6.1. The FEM included the wings, substrate, and a sphere of surrounding fluid. To reduce computing requirements, the model was bisected along the center of the length of the sensor bridge with symmetry constraints applied along this bisection.

The material properties of the substrate and sensor components were modeled as anisotropic, single-crystal silicon based on the properties of silicon used in the sensor fabrication [46]. The elasticity matrix of the material was adjusted to match the orientation of the silicon in our sensor. The fluid surrounding the sensor can be modeled by a variety of substances. However, as this sensor is intended to operate in air, the fluid sphere was modeled as air at standard temperature and pressure from the COMSOL material library. The fluid sphere was surrounded by a shell that was a perfectly matched layer of air to prevent acoustic reflections.

**Figure 4:**
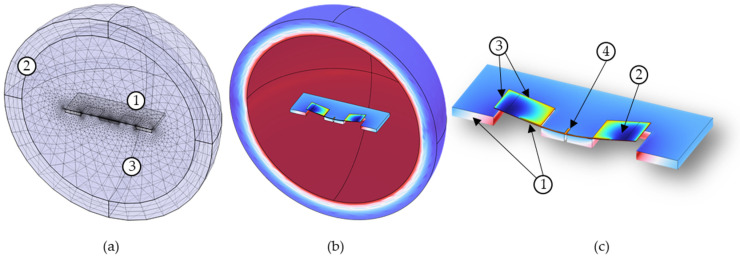
FEM study of MEMS acoustic sensor: (**a**) mesh scheme of sensor and fluid: (1) The sensor meshing utilizes a combination of free triangle mesh with swept mesh. (2) The boundary of the fluid sphere is a perfectly match layer that uses swept mesh. (3) The fluid inside the shell uses a free tetrahedral mesh; (**b**) results of a sound pressure level study showing the sensor in the bending mode; and (**c**) Detailed image of the sensor in the bending mode. Boundary conditions were applied to the model: (1) symmetry conditions along the bisected edge of the sensor and substrate, (2) boundary load conditions applied to top surfaces of wings, (3) separate boundary conditions applied to sides of wings and comb fingers along the gap between the sensor and substrate, and (4) fixed constraint attaching the torsional leg to the substrate.

The FEM included pressure acoustics and solid mechanics. Acoustic wave radiation conditions were applied to the outside edge of the sphere. Boundary loads were applied between the wings and substrate to simulate the resistance from the flow through the gaps between the sensor and substrate. Separate boundary loads were applied to the top surface of the wings to account for form friction. The acoustic wave was modeled as a background pressure field plane wave. The acoustic DOA was set using an adjustable parameter to control the wave direction. Figure 4a depicts the meshing scheme used for the sensor mode. Figure 4b shows the results of a sound-pressure-level study while depicting the sensor in the bending resonant mode. In both Figures, the fluid within the sphere is transparent for the sake of image clarity. Figure 4c depicts a detailed image of the sensor in the bending mode and highlights some of the key boundary conditions utilized in the model.

**Figure 5:**
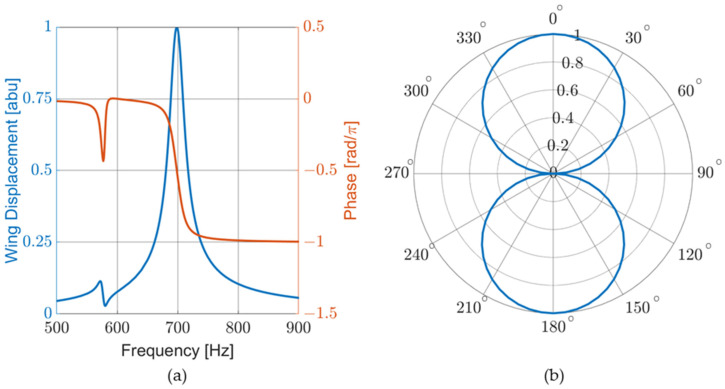
COMSOL FEM of sensor behavior: (**a**) normalized mechanical response and sensor phase response to frequency sweep. The large peak at 700 Hz corresponds to the bending mode. The phase response matches that of a simple harmonic oscillator; and (**b**) normalized mechanical response to acoustic propagation angle.

Two studies were conducted as part of the FEM analysis: a frequency sweep and a directionality analysis, where the displacement of the wing and the associated phase shift from the pressure wave were modeled. The frequency sweep was performed for a constant angle of incidence of 45 degrees, as seen in Figure 5a. In the directionality analysis, the direction of propagation of the incident wave was rotated 360 degrees in 5-degree increments around the bisection while the frequency was kept constant. The FEM shows a cosine-like response to the DOA, as seen in Figure 5b.

Electro-static effects were not modeled in the FEM analysis. Data collection on earlier generations of similar sensors showed that electrostatic forces do not appreciably affect the performance of the sensors. For this sensor, measurements were taken with the sensor electrically isolated and electrically connected to a powered PCB. No significant changes to the resonant frequency or quality factor were noted.

The FEM resonant frequency was 699 Hz with a quality factor of 33. Laser vibrometry measurements of multiple sensors showed an average bending mode frequency of 671 Hz and an average quality factor of 27. The sensor resonant frequencies ranged from 662 Hz to 679 Hz. These results indicate that there are physical effects which impact the sensor response that were not fully accounted for in the FEM, such as dimensional variation due to fabrication tolerances and actual material properties. However, the FEM, as it stands, is a useful tool for sensor design.

### 2.5. DOA Estimation

**Figure 6:**
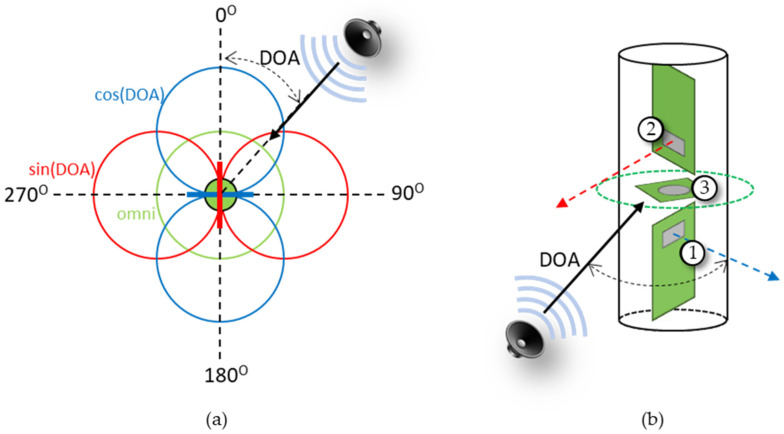
Diagram of AVS: (**a**) top-down view. The AVS can be used to determine the direction of incoming sound (DOA) and consists of a cosine sensor (blue), orthogonal sine sensor (red), and omnidirectional sensor (green); and (**b**) 3-D view: (1) cosine sensor, (2) sine sensor, and (3) omnidirectional sensor.

The MEMS acoustic sensors described in this paper were used to create an AVS array by placing two collocated MEMS sensors perpendicular to each other with an omnidirectional microphone (Knowles MEMS microphones model: SPM0687LR5H [47]) or hydrophone (Brüel & Kjær (B&K), Nærum, Denmark, Type 8103 [48]) placed between them. The DOA was measured from the normal of the reference MEMS sensor, which was designated here as the cosine sensor. The perpendicular MEMS sensor was designated as the sine sensor. A diagram of the AVS array design is shown in Figure 6a. A three-dimensional representation of how the individual sensors are arranged to form the AVS is shown in Figure 6b. The algorithm employed to calculate the DOA is explained in detail in [28] and it is given by

(10) $$DOA=atan\left(\frac{\sum \left|M_{s}V_{s}\right|sgn\left(num\right)}{\sum \left|V_{C}\right|sgn\left(den\right)}\right)\;,$$

where

(11) $$num=\sum Re\left\{M_{S}V_{S}V_{O}^{*}\right\}$$

and

(12) $$den=\sum Re\left\{V_{C}V_{O}^{*}\right\}$$

where *V_S_*, *V_C_*, and *V_O_* are the sine, cosine, and omnidirectional sensor voltage Fourier transforms. *M_S_* is a correction function that accounts for the differences in frequency response of the two MEMS directional sensors. The term sgn(.) is the sign function. Since the omnidirectional microphone is not ideal, only the phase contribution was used (sgn(*num*) and sgn(*den*) terms) to determine the quadrant.

Many underwater acoustic localization applications require the knowledge of azimuth and elevation, which was not possible with the single AVS demonstrated in this manuscript. The use of two devices orthogonally placed would solve this problem. A more elegant option being studied by our group is to use the rocking vibrational mode of the MEMS sensors, which has a sine dependence on the incoming sound, in combination with the bending motion. This would provide 3D unambiguous coverage with the same arrangement demonstrated in this paper.

## 3. Methods

### 3.1. Mechanical Sensitivity Measurements

All in-air laboratory measurements were taken inside an anechoic chamber. The chamber is made with 12-inch-thick concrete walls and is mechanically and acoustically isolated from the rest of the building housing the chamber. It is surrounded on the walls, ceiling, and floor with fiberglass wedges which absorb 99% of incident sound for frequencies greater than 100 Hz [27]. 

**Figure 7:**
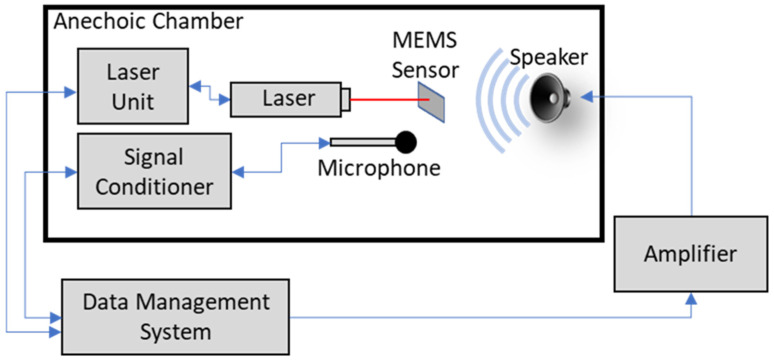
Experimental setup for mechanical sensitivity measurement.

Mechanical sensitivity measurements were taken using a laser vibrometer setup consisting of a Polytech data management system (DMS), OFV-534 laser unit, and OFV-5000 controller, as shown in Figure 7. An electrically isolated MEMS sensor was placed in a holder in the path of the laser beam. The deflection of the sensor’s wings was measured at the edge of the wing just before the beginning of the comb fingers. A frequency sweep signal was generated from the DMS through a Techron 5507 amplifier to a JBL 2450H speaker with a 2380A cone pointed toward the sensor. A Piezotronics Model 378A21 calibrated reference microphone was positioned near the MEMS sensor. The microphone signal was sent through a Piezotronics Model 482C sensor signal conditioner to the DMS. The DMS software (Polytec Vibrometer version 4.7) averaged 5 consecutive frequency sweeps to calculate a mechanical sensitivity curve, as discussed in Section 4.

### 3.2. Electrical Experimental Setup in Air

**Figure 8:**
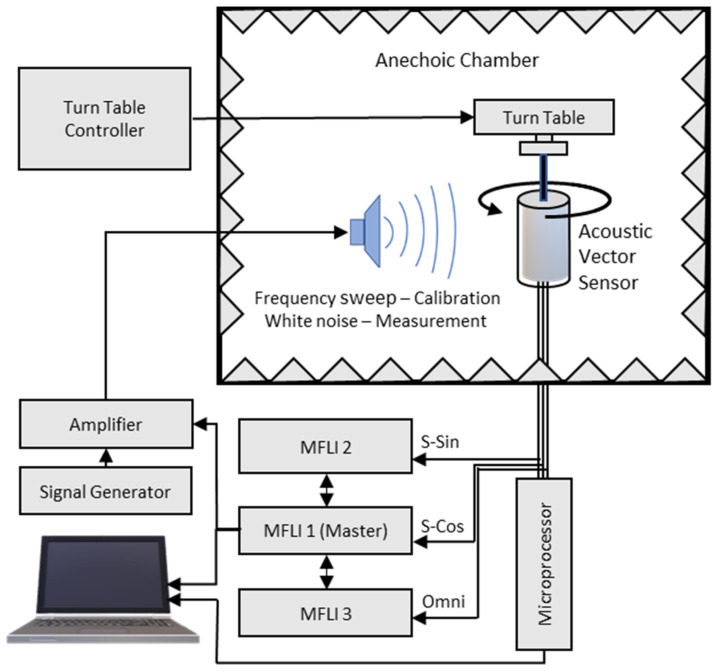
Schematic diagram of the experimental setup for AVS calibration and DOA estimation.

Electrical sensitivity and directionality measurements were taken in an anechoic chamber with the sensor electrically connected to a PCB. The MEMS sensors (either individually or in an AVS configuration) were mounted on a B&K Model 5960 turntable, operated by a B&K Type 5997 turntable controller. A rubber dampening device was installed between the sensor and turntable to reduce mechanical coupling. An acoustic signal generated by a Zurich Instruments Multifunction Lock-in Amplifier (MFLI) was sent to a Techron 5507 amplifier to a JBL 2450H speaker with a 2380A cone, which was pointed toward the AVS. Parallel signals from each sensor were sent to individual MFLIs and to a microprocessor (which calculated the DOA). An Agilent 33220A waveform generator was used in conjunction with the MFLI to generate broadband white noise. The experimental setup is shown in Figure 8.

Electrical sensitivity measurements were made with the sensor facing the speaker (DOA = 0 degrees), while the speaker produced a frequency sweep generated by the MFLI. The output of the sensor was sent to the MFLI. A Piezotronics Model 378A21 calibrated reference microphone was positioned near the MEMS sensor during the frequency sweeps. The microphone signal was sent through a Piezotronics Model 482C sensor signal conditioner (with a gain applied such that the microphone signal corresponded to the pressure) to a separate MFLI. These signals were divided to generate the electrical acoustic sensitivity of the sensor, as discussed in Section 4.

Sensor directionality was measured with single tones (near sensor resonance) generated by the MFLI. The turntable was rotated at a constant angular velocity through a 360-degree circle. The sensor output was sent to the MFLI, which continuously recorded sensor output as a function of time during the rotation. The time corresponded to the DOA angle and a radial plot of sensor directionality was generated, as in Figure 11b.

Prior to an AVS being used to calculate DOA, it must be calibrated. To calibrate the AVS, the turntable was rotated such that the DOA was set to 45 degrees. The signal generator produced a frequency sweep. The outputs of each MEMS sensor and omnidirectional microphone were sent through a microprocessor control box to a computer. A computer program received the output from the three sensors and computed the correction function, *M_S_*. This correction function was ingested in the arctangent estimator algorithm, which was run by a Teensy 4.0 microprocessor in the control box.

To characterize the AVS performance, the AVS was exposed to various acoustic signals (e.g., single tones, white noise, gunshot recordings, drone recordings). The turntable was adjusted to a known DOA value, and the microprocessor was triggered. The microprocessor calculated the DOA. The actual and calculated DOA were recorded. The turntable was then rotated to a new DOA value and the process was repeated.

### 3.3. Field Experimental Setup

**Figure 9:**
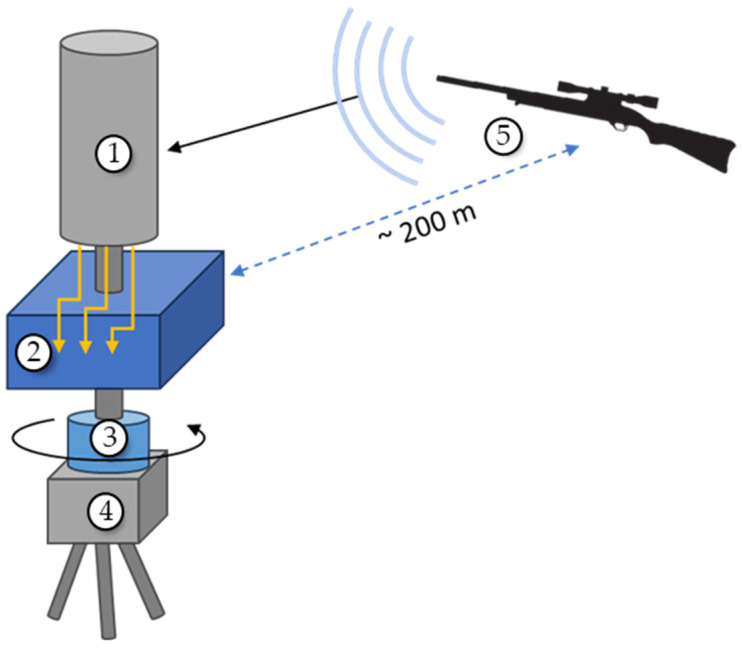
Schematic of field AVS DOA measurement setup: (1) AVS, (2) sensor signals connect to control box containing a microprocessor, (3) rotating mount, (4) tripod, and (5) rifle (sound source).

AVSs were operated in the field to measure DOA accuracy when exposed to actual gunshots. A node consisting of the AVS (encased in a protective housing and dust cover) and microprocessor control box were placed on an Edelkrone Pan Pro motorized single-axis rotation device mounted on a tripod, as shown schematically in Figure 9. The rotation device allowed for precise DOA angles to be set and allowed for continuous rotation. The AVS was adjusted to point towards the location of the shooter (approximately 200 m away), the shooter would fire their weapon, the AVS would calculate the DOA to the gunshot. Both the actual and calculated DOAs were recorded. The AVS was rotated to a new DOA and the process was repeated. Various rifles and handguns were used for separate DOA measurements.

### 3.4. Experimental Setup under Water

Individual underwater MEMS sensors were measured in the SWT, which is an aluminum tube with an outer diameter of 30.5 cm (12 in), an inner diameter of 25.4 cm (10 in) and is 61.0 cm (24 in) tall. An Electro Voice UW30 underwater loudspeaker was placed at the bottom of the SWT on vibration-damping material. The SWT was filled with water such that the water surface was approximately 33 cm (13 in) above the top of the speaker. The sensor, contained in a watertight housing, was held on a suspended rod with a rotating mechanism, which allowed for the DOA of the sensor to be adjusted.

An acoustic signal was generated by an MFLI and sent to a Hewlett Packard 467A amplifier, which powered the speaker. Acoustic measurements were taken by a B&K Type 8103 hydrophone. The hydrophone output was sent to a Stanford Research Systems Model SR560 low-noise preamp and then to an MFLI. The MEMS sensor output was sent directly to an MFLI.

Electric acoustic sensitivity measurements were made with the sensor facing the speaker (DOA = 0 degrees). A frequency sweep was generated by the MFLI. Outputs from the hydrophone and MEMS sensor were sent to MFLIs. The output of the hydrophone was used to calculate the acoustic pressure, which was then used with the MEMS sensor output to determine sensitivity.

The directionality measurements for the underwater sensor were taken in the similar manner to the in-air directionality data. Directional measurements were made by connecting the Edelkrone Pan Pro rotation device to the rotator at the end of the suspension rod. The MFLI generated a single tone while the sensor was rotated at a constant angular velocity through 360 degrees. 

Underwater AVS measurements were taken at the TRANSDEC anechoic pool. The AVS was suspended on a rotating pole 2 m away from a Lubell Labs VC2C underwater speaker. Both the sensor and speaker were 6 m from the surface and bottom of the pool. As with in-air AVS data collection, the MEMS sensor outputs were sent to the microprocessor control box. Instead of a microphone, the underwater AVS used a B&K Type 8103 hydrophone as the omnidirectional sensor. The hydrophone output was sent to a Stanford Research Systems Model SR560 low-noise preamp and then to the microprocessor. The rotating pole was adjusted to set the AVS to a known DOA while a constant tone was played by the underwater speaker. The microprocessor was triggered, and the DOA was calculated. The actual and calculated DOAs were recorded. Then, the pole was rotated to set a new DOA and the process was repeated.

## 4. Experimental Results

### 4.1. Operation in Air

**Figure 10:**
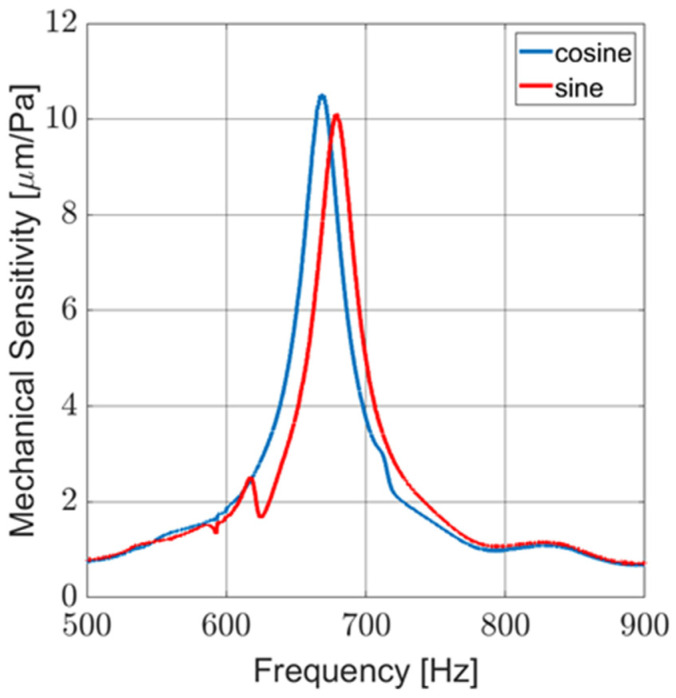
Laser vibrometry measurement of mechanical sensitivity of typical MEMS sensors used in AVS.

Laser vibrometry measurements were taken for all MEMS sensors. Wing displacement was measured and compared against the acoustic pressure of a frequency sweep to determine the mechanical sensitivity (μm/Pa). These measurements were conducted in an anechoic chamber and the response of a typical pair of sensors is shown in Figure 10. Note the mismatch of the resonant peaks. This mismatch is compensated by the correction function, *M_S_*, in (10) and (11).

**Figure 11:**
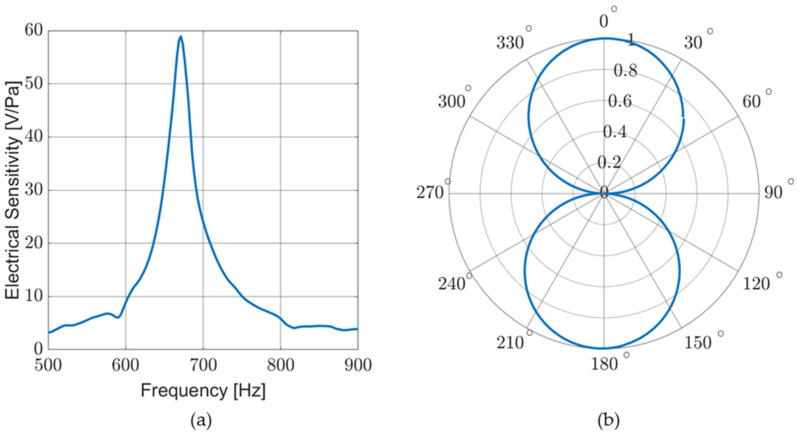
Sensor response in air: (**a**) electrical sensitivity measured for a frequency sweep. Electrical sensitivity corresponds to mechanical sensitivity; and (**b**) normalized sensor response to a single tone (near resonance) while rotating sensor to sweep DOA.

Electrical sensitivity (V/Pa), as well as directionality, were measured in an anechoic chamber. Figure 11a shows the response of a sensor in air for a frequency sweep conducted with the sensor facing the sound source (DOA ≈ 0 degrees). Figure 11b shows the cosine-like response of the sensor as it is rotated near an acoustic source producing a single tone near resonance. Although not pictured, additional directional response measurements were taken for a variety of single-tone frequencies and broadband stimuli with similar results.

**Figure 12:**
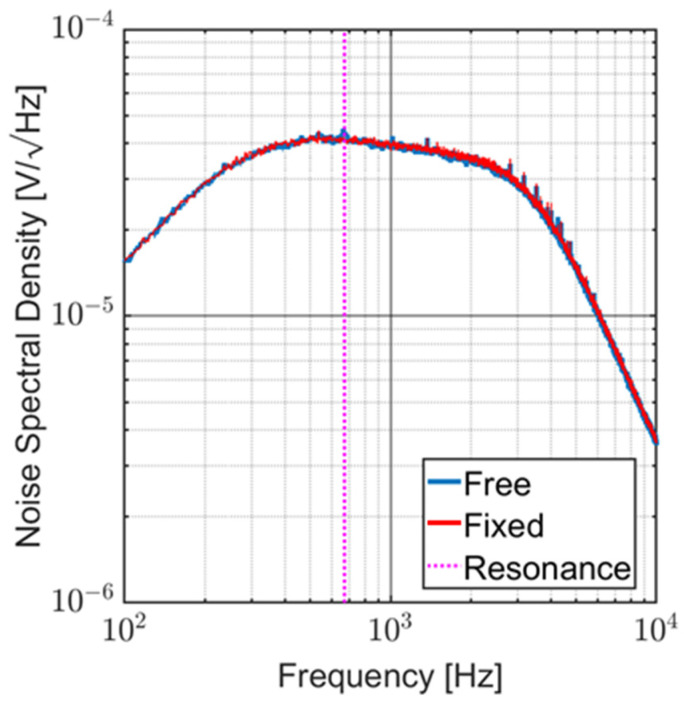
Noise spectral density of typical MEMs sensor. It is possible to notice that electronic noise is predominant, and the natural vibrations of the sensor are not captured by the circuit.

Noise measurements were performed in an anechoic chamber with the sensor readout electronically powered and with no acoustic stimuli in two different configurations. First, the sensor wings were free. Then, the sensor wings were cemented to the substrate to prevent their natural vibration. Figure 12 shows the noise spectral density (V/√Hz) over a 100 Hz to 10 kHz band. Since both curves are coincident, it is possible to infer that the electronic noise is predominant and that the natural vibrations of the sensor are not captured by the circuit. The SNR at resonance for an acoustic stimulus of 1 Pa over the 3 kHz band of the circuit is approximately 88 dB. Since the sensor is meant to operate at resonance, the Sallen–Key filter stages (Figure 2a) can be designed for a much narrower passband. With a 120 Hz bandwidth, the SNR at resonance becomes greater than 102 dB.

Prior to use in the field, the AVSs were calibrated in an anechoic chamber. This process is described in Section 3 and in [28]. The frequency response of the sine and cosine sensors are measured (*V_C_* and *V_S_*) with the DOA set to 45 degrees. Ideally, the frequency responses of both sensors at 45 degrees should be the same, however, since they are not, *M_S_* = *V_C_*/*V_S_* is applied to the sine signal. The signal processing electronics calculate the DOA, using (10), as the AVS is rotated.

**Figure 13:**
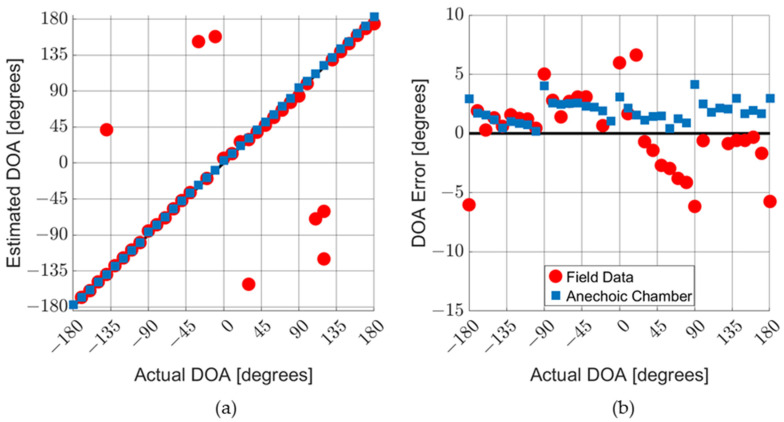
AVS characterization in air with gunshots. AVS was exposed to gunshot audio recording in an anechoic chamber and actual gun fire, from same time of weapon, in the field: (**a**) comparison of the actual DOA with the estimated DOA; and (**b**) detailed graph of DOA errors. Outlier data points are not shown on the DOA error graph.

**Figure 14:**
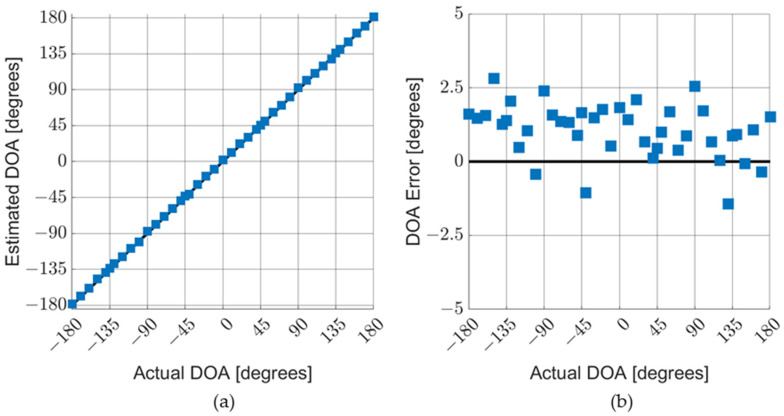
AVS characterization in air with drone sounds. The AVS was exposed to audio recordings of a four-rotor drone: (**a**) comparison of actual DOA with estimated DOA; and (**b**) detailed graph of DOA errors.

Measurements were taken in an anechoic chamber and in the field for various acoustic sources, particularly gunfire and small multi-rotor aircraft (drones). Figure 13a shows the results for the DOA characterization measurements of an AVS. The blue squares represent data taken in an anechoic chamber with the AVS exposed to an audio recording of rifle fire. The red circles represent data taken in the field with the AVS exposed to actual fire from the same type of rifle. Figure 13b shows the detection error, which was calculated as the difference between the measured and actual DOA. Figure 14a,b shows the results for the similar data collection of an AVS operating in an anechoic chamber exposed to audio recordings of a typical four-rotor drone in flight.

The anechoic chamber gunshot data and drone data show a systematic error offset to the positive side. This indicates a misalignment of the turntable used to rotate the AVS when setting the DOA. Field data typically have larger errors due to reverberations, acoustic reflections, and background noise. In field experiments, the AVS occasionally collects data on gunshot sounds that reflect off nearby structures, which is the case with the outlier points in Figure 13a.

The DOA errors for both drones and gunshots (for data taken in an anechoic chamber) fall within a 0-to-5-degree range (which corresponds to a ±2.5-degree range when misalignment is corrected for). It should be noted that the drone sound used to characterize the AVS (as shown in Figure 14) aligned well the MEMS sensors. Different drone sounds produce DOA errors of up to ±5 degrees. The tonal nature of the acoustic signatures of drones tends to lead to larger DOA errors than gunshots.

**Figure 15:**
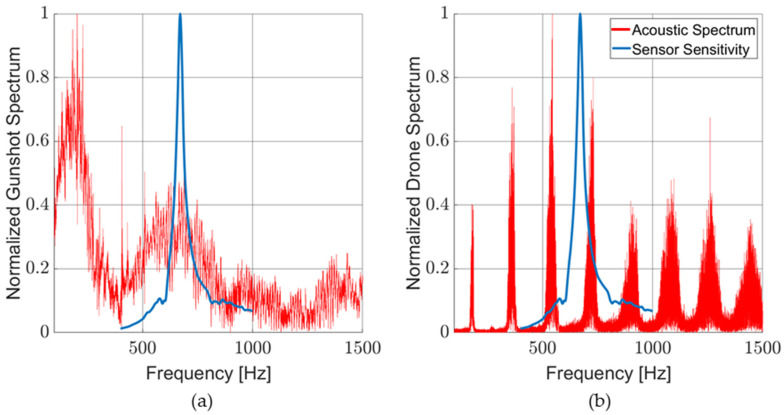
Acoustic spectrum of common sound sources: (**a**) typical gunshot acoustic spectrum with MEMS sensor sensitivity overlay; and (**b**) typical drone acoustic spectrum with MEMS sensor sensitivity overlay.

Figure 15a,b shows the typical acoustic signatures of pistol gunfire and a small four-rotor drone, respectively, measured with a broadband microphone (Piezotronics Model 378A21). The acoustic signatures are normalized to their maximum values and plotted against the normalized acoustic sensitivity of a typical MEMS sensor. Gunshot sounds, while bursts, are typically broadband in the frequency range interrogated by this sensor. Drone sounds exhibit high peaks at the harmonics of the blade passing frequency (BPF). These tones differ from drone to drone. Note that in Figure 15b, the MEMS sensor peak sensitivity is misaligned with the drone BPF harmonic at around 700 Hz. If this signature is known, a MEMS sensor can be designed to align with a specific harmonic. This will maximize drone detection while filtering out other acoustic sources. More details on these types of acoustic sources can be found in [49,50,51,52,53].

### 4.2. Operations Underwater

**Figure 16:**
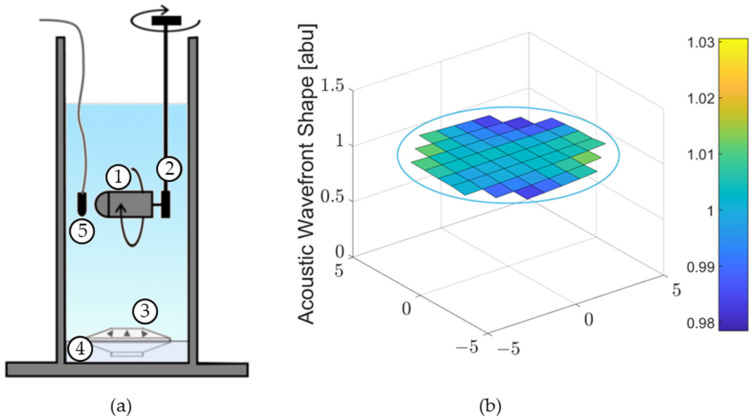
(**a**) Standing wave tube experimental setup: (1) sensor in housing, (2) rotating mechanism, (3) underwater speaker, (4) sound damping material, and (5) reference hydrophone; and (**b**) acoustic pressure wave front measured at 640 Hz, 6 in depth. The blue ring represents the 10-inch inner diameter of the tube.

Data collection for underwater sensor operations was conducted, for the most part, in a vertical, water-filled, standing wave tube (SWT). A diagram of the experimental setup is shown in Figure 16a. A detailed description of the experiment can be found in Section 3. The acoustic properties of the SWT were characterized to verify that a flat acoustic wave front is produced in the section of the tube where the sensor is operated. Figure 16b shows the acoustic pressure value at a depth of 15.2 cm (6 in) below the surface for a constant frequency and speaker voltage. This depth corresponds to the depth of the sensor during data collection. The data show that the wave front is essentially flat and mimics a plane wave. The average pressure is 9.9 Pa with a standard deviation of 0.1 Pa. The most significant wavefront distortion occurs near the tube wall, with a maximum deviation of 3% from the average. During data collection, the sensor was placed in the center of the tube where the wavefront is the flattest.

**Figure 17:**
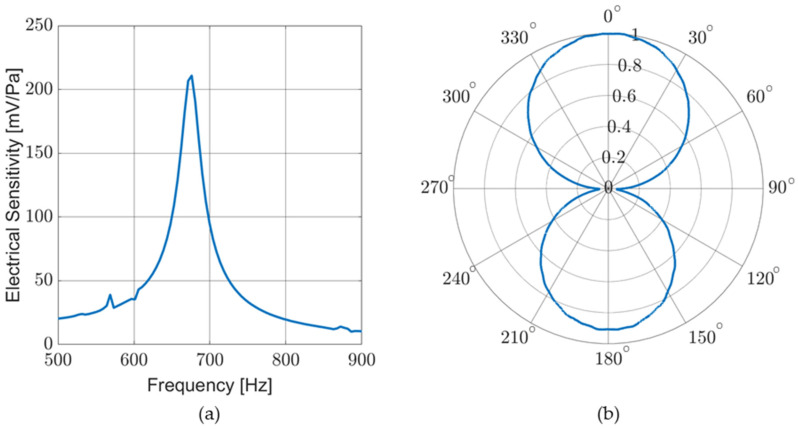
Sensor response in standing wave tube: (**a**) electrical sensitivity measured during a frequency sweep; and (**b**) normalized sensor response to a single tone (near resonance) while rotating sensor to sweep DOA.

The sensitivity and DOA response of the underwater sensor were measured in a similar manner to the in-air measurements. Figure 17 shows typical MEMS sensor characteristics when measured in the SWT. While there is no significant difference in the frequency response as compared to in-air frequency sweeps, there is a noticeable difference in lobe size between the front and back of the sensor. This is likely caused by the influence of the sensor housing. This lobe mismatch was observed for all underwater DOA measurements in the SWT.

**Figure 18:**
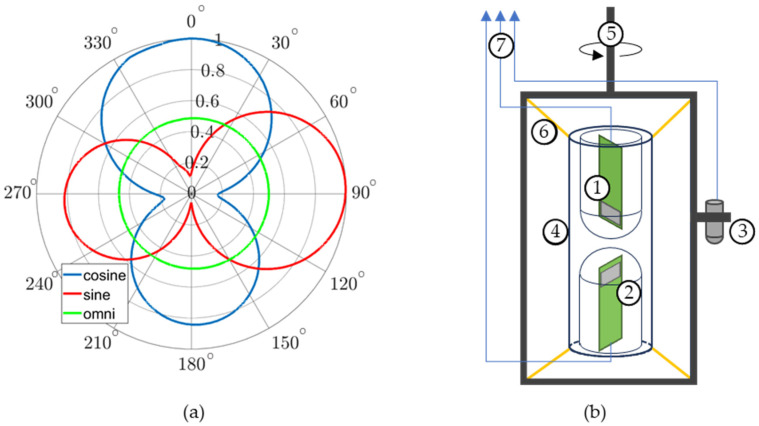
(**a**) Measured directionality response of AVS to 670 Hz tone at TRANSEC. Sine and cosine sensor responses are normalized to the maximum cosine response. The omni response is significantly less than the MEMS sensor but is enlarged to show the shape of its directionality; and (**b**) diagram of underwater AVS setup: (1) sine sensor in underwater housing, (2) cosine sensor in underwater housing, (3) omnidirectional hydrophone, (4) sensor alignment tube, (5) rotating mounting frame, (6) elastic bands connecting sensor housing to rotating frame, and (7) output signals to microprocessor.

**Figure 19:**
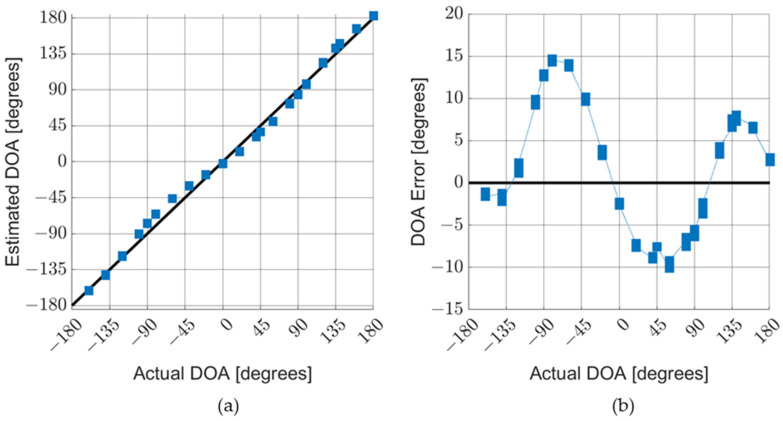
AVS underwater characterization. AVS was exposed to a 670 Hz tone in an anechoic pool: (**a**) comparison of actual DOA with estimated DOA; and (**b**) detailed graph of DOA errors.

Underwater AVS measurements were taken at the Transducer Evaluation Center (TRANSDEC) which is a six-million-gallon Navy facility designed to test underwater acoustic devices. The MEMS sensors were individually evaluated and the AVSs were calibrated at TRANSEC prior to conducting the DOA measurements. Figure 18a shows the directionality of each sensor in the AVS. A reduced back lobe was observed similar to the data obtained in the SWT. Figure 18b shows a diagram of the underwater AVS setup. Figure 19a,b shows the results of a DOA characterization for an AVS stimulated by a 670 Hz single tone. The average of the DOA error magnitude is approximately 6.7 degrees.

It is noticeable that, even though the estimator used underwater was the same as that used for in-air operation, the error is higher. Possible sources of this error included surface reflections and effects from the AVS mounting apparatus. Surface reflections were observed for the frequency ranges investigated with this AVS. As noted above, the back lobes of the MEMS sensors are smaller than the front lobes when operated underwater. This phenomenon was seen both in the SWT and the anechoic pool using different mounting schemes. Therefore, the underwater housing is likely affecting the sensor response.

A sinusoid-like shape to the DOA error is observable for the underwater sensor. There are two primary causes of a sinusoid-like DOA error: amplitude and phase mismatches between the two MEMS sensors. The minimum error amplitudes are seen at cardinal angles (i.e., 0, ±90, and 180 degrees), where either vs. or *V_C_* is near zero and the algorithm is less sensitive to fluctuations. The maximum error magnitudes are near the center of each quadrant (i.e., ±45 and ±135 degrees), where the amplitude mismatch is most significant. Phase mismatches (for small phase angles) lead to a different sinusoid-like DOA error centered at approximately half of the mismatch in the phase. The correction factor, *M_S_*, compensates for the differences between vs. and *V_C_*. However, vs. and *V_C_* are determined by applying a Fourier transform to the sine and cosine sensor signals, respectively. Consequently, there are limitations on the frequency bin size of vs. and *V_C_*. Broadband signals (e.g., white noise, gunshots) are naturally integrated across these frequency bins and the error is minimized. Single-tone sound sources do not benefit from this phenomenon and consequently show larger errors.

## 5. Discussion/Conclusions

This paper describes the design and experimental characterization of directional acoustic MEMS sensors and an AVS comprised of two collocated and orthogonally aligned sensors in combination with a commercial omnidirectional microphone (or hydrophone). The AVS can determine in-plane acoustic DOA over a non-ambiguous 360 degrees. This AVS is meant to operate near resonance and to be used in the air and underwater. The configuration of the MEMS sensors, AVS design, and algorithms used make this a novel approach to using *Ormia*-inspired MEMS acoustic sensors to determine acoustic DOA.

The small size, light weight, and low power requirements of the MEMS acoustic sensor and associated AVS show potential for their use as a man-portable sensor for detecting and locating acoustic contacts of interest. The MEMS acoustic sensor demonstrates very high SNR near resonance. This makes the sensor ideal for detecting quiet and distant acoustic targets of interest. The resonant frequency of the MEMS sensor is based on the physical characteristics of the sensor (e.g., bridge and wing size) allowing for bespoke sensors to be designed for acoustic targets that emit specific acoustic tones.

### 5.1. Sensor Performance

The maximum SNR for this MEMS sensor was determined to be 88 dB with a corresponding maximum sensitivity of −84.6 dB re 1 V/μPa (59 V/Pa). The sensor demonstrates a cosine-like acoustic directionality for the bending eigenmode. This sensitivity is significantly larger than comparable MEMS acoustic sensors, as seen in Table 2. Note that the SNR reported in [27] is over the frequency band of resonance of the sensor (120 Hz bandwidth). The SNR reported for this sensor was calculated over the frequency range of its associated circuit (0 to 3 kHz). When calculating noise only about this sensor’s resonant peak (120 Hz bandwidth), it would have an SNR of approximately 102 dB.

### 5.2. AVS Performance

The accuracy of the AVS was measured both in the lab and in the field for use in both the air and underwater. While calculating the DOA of gunshots in the field, the average best case AVS accuracy was approximately 3.5 degrees. AVS accuracy, measured in the lab, was determined to be less than an average of 2 degrees over a 360-degree arc. This performance is an improvement on previous laboratory measurements reported by this group of 3.4-degree accuracy over a ±60-degree arc [23]. The performance details of comparable AVS designs are presented in Table 3. Note that the performance data for the AVS presented in this paper were collected in the field with actual gunfire. The tabulated data for all other AVS designs were taken in an anechoic chamber.

These results indicate the great potential of this type of MEMS sensor for DOA determination in multiple domains. The specific characteristics and figures of performance can be modified by design, according to the application demands. Future work includes the development of multi-resonance MEMS sensors for use in detecting very quiet broadband acoustic sources.

## Figures and Tables

**Table 1:** Modeled sensor behavior compared against sensor behavior measured in laboratory. The error of the 7-1 analytical model is notably larger than the 7-3 error. A contributing factor is the characteristic length, b, used to calculate the Reynolds number in (3). The analytical model assumes that wing width is sufficient to use as the characteristic length, b. However, the ratio of the wing width to bridge width for the 7-1 design is an order of magnitude greater than it is for the 7-3 design.

Model	Key Design Parameters [μm]	Resonant Freq Model [Hz]	Q_1_	Q_2_	Quality Factor Model	Resonant Freq Measured [Hz]	Quality Factor Measured
7-1 Air	Wing Width = 3000 Wing Length = 1450 Bridge Width = 80 Bridge Length = 1960	607	26	162	22	664	27
7-3 Air	Wing Width = 2400 Wing Length = 1595 Bridge Width = 500 Bridge Length = 1400	2345	53	1017	50	2340	59
7-3 Oil		435	22	48	15	440	4

**Table 2:** Comparison of MEMS sensor performance.

	This Sensor(7-1)	Double-Wing MEMS [27]	Double-Wing Design [16]	16 Cantilever Beam Design [54]	Circular Membrane Design [12]	8 Cantilever Beam Design [55]
Sensitivity	59 V/Pa	13 V/Pa	110.5 mV/Pa	70.8 mV/Pa	4.36 mV/Pa	1.67 mV/Pa
SNR	88 dB [102 dB]	91 dB	71.3 dB	51 dB	66.77 dB	Not Discussed

**Table 3:** Comparison of AVS performance.

	This Sensor (7-1)	Three-Sensor Array [19]	Double Diaphragm Design [15]	2 Sensor Array [13]	Canted Double-Wing AVS [23]
DOA Arc	360°	360° ^1^	180°	90°	±60°
Average DOA Accuracy	3.5°	2°	2.6°	Not Specified	3.4°

^1^ DOA determined with a priori knowledge.

## Data Availability

The data that support the findings of this study are available from the corresponding author upon reasonable request.
